# Telemedicine and the assessment of clinician time: a scoping review

**DOI:** 10.1017/S0266462323002830

**Published:** 2023-12-15

**Authors:** Kristian Kidholm, Lise Kvistgaard Jensen, Minna Johansson, Victor M. Montori

**Affiliations:** 1 Center for Innovative Medical Technology, Odense University Hospital and University of Southern Denmark, Denmark; 2 Global Center for Sustainable Healthcare, School of Public Health and Community Medicine, Sahlgrenska Academy, University of Gothenburg, Gothenburg, Sweden; 3 Department of Medicine, Mayo Clinic, Knowledge and Evaluation Research Unit, Rochester, MN, USA

**Keywords:** telemedicine, clinician time, workload, organisational impact, digital technologies

## Abstract

**Objectives:**

Telemedicine may improve healthcare access and efficiency if it demands less clinician time than usual care. We sought to describe the degree to which telemedicine trials assess the effect of telemedicine on clinicians’ time and to discuss how including the time needed to treat (TNT) in health technology assessment (HTA) could affect the design of telemedicine services and studies.

**Methods:**

We conducted a scoping review by searching clinicaltrials.gov using the search term “telemedicine” and limiting results to randomized trials or observational studies registered between January 2012 and October 2023. We then reviewed trial registration data to determine if any of the outcomes assessed in the trials measured effect on clinicians’ time.

**Results:**

We found 113 studies and of these 78 studies of telemedicine met the inclusion criteria and were included. Nine (12 percent) of the 78 studies had some measure of clinician time as a primary outcome, and 11 (14 percent) as a secondary outcome. Four studies compared direct measures of TNT with telemedicine versus usual care, but no statistically significant difference was found. Of the sixteen studies including indirect measures of clinician time, thirteen found no significant effects, two found a statistically significant reduction, and one found a statistically significant increase.

**Conclusions:**

This scoping review found that clinician time is not commonly measured in studies of telemedicine interventions. Attention to telemedicine’s TNT in clinical studies and HTAs of telemedicine in practice may bring attention to the organization of clinical workflows and increase the value of telemedicine.

## Introduction

Telemedicine, defined as the use of information and communication technologies to deliver healthcare services at a distance, increased during the COVID-19 pandemic to offer access to healthcare without direct contact. In the years 2020–2022, video calls and home monitoring of patients with chronic disease increased internationally (1). In Ontario, Canada, remote visits increased from 2 percent of ambulatory visits in the second quarter of 2019 to 71 percent in the second quarter of 2020 (2). In the United States, data from the Centers for Medicare and Medicaid Services showed an increase across nearly all medical specialties in weekly remote care – for example, video and audio-only visits, care chat, secure e-mail, and telemonitoring transfer of remote vital data – from 13,000 before the pandemic to 1.7 million in April 2020 (3). In China, 95 percent of 148 surveyed physicians from 57 hospitals in 16 provinces adopted telemedicine systems during the pandemic (4); In the United Kingdom, remote consulting increased in primary care from 30 to 89 percent in the months immediately before and after the start of lockdown and use of short message service from general practitioner to patients more than tripled from 23 messages per 1000 patients in July 2019 to 76 messages per 1000 patients in July 2020 (5).

The pandemic has brought attention to a substantial healthcare force deficit caused by demographic challenges, clinicians’ increased risk of exposure to infections, increased patient demand for care, and clinician burnout and practice exit (6;7). The UN High-level Commission on Health Employment and Economic Growth (8) and the WHO’s National Workforce Accounts have estimated a global health workforce shortage of 15.4 million health workers in 2020 (9) distributed unequally, affecting African and Eastern Mediterranean countries worse than the countries in the Western Pacific Region.

Experts have argued that telemedicine may favorably affect the workload of health professionals and reduce clinician time spent on delivering care. For example, a letter to the editor in the *Journal of Clinical Virology* argues that telemedicine can be used to maintain healthcare providers’ well-being by reducing unnecessary patient visits, promoting self-quarantine, and reducing emergency department overuse thus reducing the workload of physicians and minimizing exposure risk for healthcare workers (10). Some evidence supports these speculations. For example, a systematic review including fourteen studies of the effectiveness of tele-triage found that using telehealth to triage patients could reduce the number of unnecessary emergency room visits by 1.2–22.2 percent and reduce clinician workload (11). Were telemedicine to reduce the time needed to treat, it could represent an important solution to the problems stemming from post-pandemic workforce constraints and increased healthcare demand.

We recently proposed that policymakers should estimate the time needed to treat (TNT) to clarify the effect of translating recommended care into practice on clinicians’ time and on the resulting opportunity costs for other patients and problems (12).

Several frameworks and guidelines for health technology assessment (HTA) and economic evaluation recommend the assessment of the effect of telemedicine on system performance, such as productivity and efficiency, but do not specify the need to assess clinician’s time (13;14). One HTA-based model, the model for assessment of telemedicine (MAST) (15), specifies that resources used when delivering the telemedicine application (e.g., clinician time) should be included in the estimated costs. MAST also includes assessment of the organizational aspects of telemedicine defined as “what kind of resources have to be mobilized and organized when implementing a new technology, and what kind of changes or consequences the use can further produce in the organization” (15). However, a review of empirical health technology assessments using the MAST framework (16) has found only one study assessing the effect of telemedicine on workload, and time spent by physicians and nurses (17). This inattention to the effect of telemedicine on clinicians’ time may be due to a lack of evidence about this effect in clinical trials of telemedicine interventions. The objective of this article is to describe the degree to which clinical trials of telemedicine interventions have assessed their impact on clinicians’ time as an outcome. We then discuss how including TNT in HTA of telemedicine could affect the design of telemedicine services and studies. It is our hope that our study will emphasize the need for assessment of the effects of telemedicine on TNT in clinical studies and HTA, and thereby improve the possibilities to identify technologies that reduce the problems related to staff shortage in the healthcare systems.

## Methods

### Data sources

To identify how and to what extent studies of telemedicine interventions have included clinician time as a primary or secondary outcome, we carried out a review of studies of telemedicine on www.clinicaltrials.gov (18). The database is maintained by the United States National Library of Medicine and the National Institutes of Health and is the largest database of clinical studies in the world. The database includes both publicly and privately funded clinical studies, and the structured description of the studies includes detailed information on the intervention, study design, and primary and secondary outcome measures. Thus, by reviewing this database information about all outcome measures included in each of the studies could be collected.

Two authors (K.K. and L.K.J.) searched the database using the search term “telemedicine” and limited results to studies registered between January 1, 2012 and December 1, 2022 and tagged as completed or terminated studies with results.

### Study selection

Eligible studies were randomized trials or observational studies with a comparison group of telemedicine interventions, that is the delivery of healthcare services through the use of information and communication technologies at a distance. Thus, the inclusion criteria are only regarding the study design and the type of clinical intervention. Two observers (K.K. and L. K.J.) working independently and in duplicate, evaluated the eligibility of each registration resolving disagreement by consensus after a joint review of the registration.

### Data collection and analysis

Two observers (K.K. and L. K.J.) extracted from each trial registration record the trial primary outcomes and whether the secondary outcomes included direct or indirect measures of clinician time, and trial results for these outcomes. To be inclusive, we considered outcomes that assessed the direct effect of telemedicine on clinician time, that is, the time (minutes per patient and/or minutes for the whole population of patients) the clinician spent providing the telemedicine service and also outcomes assessing the indirect effect on the clinician time in the healthcare system in general, for instance considering the effect of telemedicine on the number of outpatient visits, hospital admissions or bed days. Related publications of the trial (indexed automatically by the registry) were reviewed whenever clinicians’ time was a trial outcome but the record did not include results.

## Results

**Figure 1.**
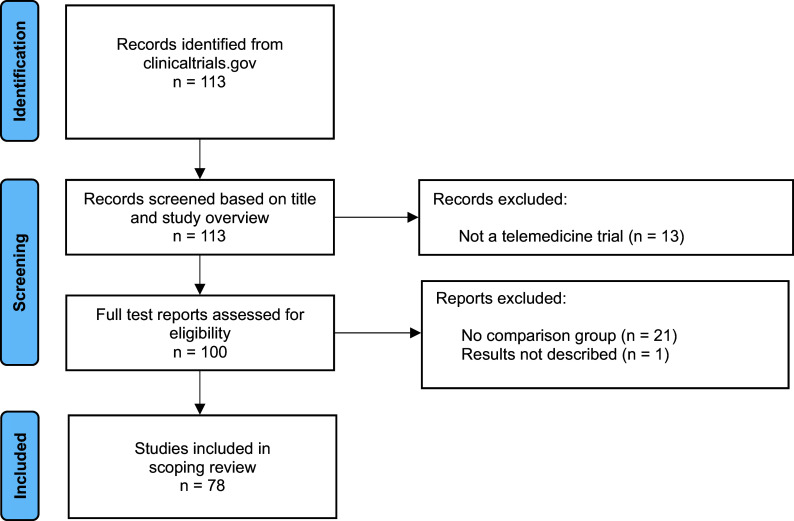
PRISMA flow chart of included studies.

We identified 113 clinicaltrials.gov records in the search. Of these, thirteen records were excluded because the studies did not match our inclusion criteria, for example, they were testing how to implement telemedicine and not the effect of a telemedicine service. Based on the full-text report, an additional twenty-two records were excluded for various reasons (Figure 1), while seventy-eight records met the inclusion criteria and were included in the review (Table 1). Of the seventy-eight records, seventy-five were randomized controlled trials and three were observational studies with a control group.

**Table 1.** Records identified in clinicaltrials.gov

Title of study	Primært outcome described on clinicaltrials.gov	Secondary outcome including clinician time described on clinicaltrials.gov	Results regarding clinician time reported on clinicaltrials.gov or links to publications	URL
Stroke telemedicine outpatient prevention program for blood pressure reduction	Blood pressure	Acute healthcare utilization	No statistical significant difference was found	https://ClinicalTrials.gov/show/NCT03923790
Can novel telemedicine tools reduce disparities related to early identification of autism	Accurate diagnosis	Not included	Not measured	https://ClinicalTrials.gov/show/NCT03847337
Telemedicine in patients with inflammatory bowel disease (TELE-IBD)	Disease activity, QoL	Healthcare utilization	No statistical significant difference was found	https://ClinicalTrials.gov/show/NCT01692743
Telemedicine management of chronic insomnia	Insomnia severity index score	Not included	Not reported	https://ClinicalTrials.gov/show/NCT01686438
Telemedicine intervention to improve cognitive function	Alzheimer disease assessment scale	Not included	Not reported	https://ClinicalTrials.gov/show/NCT02248649
Utilizing telemedicine for delivery of postoperative care	General satisfaction	Actual visit time, visits to AD, phone calls, time dedicated by patient to complete visit	No statistical significant difference was found	https://ClinicalTrials.gov/show/NCT04348357
Telemedicine intervention to improve physical function	Patient falls	Not included	Not reported	https://ClinicalTrials.gov/show/NCT01639469
Philadelphia telemedicine glaucoma detection and follow-up study	Detection of glaucoma, confirmation by MD	Not included	Not reported	https://ClinicalTrials.gov/show/NCT02390245
Effect of mobile phone telemedicine on diabetes care	Satisfaction and usability	No secondary outcomes	Not reported	https://ClinicalTrials.gov/show/NCT01698008
School-based telemedicine enhanced asthma management	Symptom-free days	No secondary outcomes	Not reported	https://ClinicalTrials.gov/show/NCT01650844
Study of telemedicine stress management and lifestyle group intervention for HCV patients	Proportion of patients consenting to participate, retained and completed, and PROMIS health score	Not included	Not reported	https://ClinicalTrials.gov/show/NCT04198584
Telehealth after stroke care: integrated multidisciplinary access to post-stroke care	Change in BP, percentage completing video consultation	Not included	Not reported	https://ClinicalTrials.gov/show/NCT04640519
Telehealth-based strategies to increase oral chemotherapeutic agent medication adherence	Medication adherence	Not included	Not reported	https://ClinicalTrials.gov/show/NCT02543723
Evaluating the value of telehealth for care of children with medical complexity	Hospital day length of stay	Not included	No statistical significant difference was found	https://ClinicalTrials.gov/show/NCT02849938
Telehealth management in HF disparity patients	Hospitalizations, ED visits	Not included	No statistical significant difference was found	https://ClinicalTrials.gov/show/NCT02196922
Care coordination/home telehealth to safeguard care in CKD	Number of safety events	Hospitalizations	No statistical significant difference was found	https://ClinicalTrials.gov/show/NCT03038126
Smart telehealth exercise intervention to reduce COPD readmissions	Readmission	Not included	No statistical significant difference was found	https://ClinicalTrials.gov/show/NCT03089853
Telehealth cognitive behavioral therapy for depression in Parkinson’s disease (PD)	Depression scale	Not included	Not reported	https://ClinicalTrials.gov/show/NCT02475954
Perioperative post-prostatectomy incontinence home telehealth program	Time to continence	Not included	Not reported	https://ClinicalTrials.gov/show/NCT01960998
Tele-health electronic monitoring to reduce post discharge complications and surgical site infections	Readmission, wound infection	Nursing home visits	No statistical significant difference was found	https://ClinicalTrials.gov/show/NCT02767011
Telehealth-based resistance training intervention for endometrial cancer survivors	Recruitment, completing exercise, averse events	Not included	Not reported	https://ClinicalTrials.gov/show/NCT03722030
Encouraging patient-centered communication in clinical video telehealth visits	HgbA1c, self-efficacy, adherence, patient perception of communication, empathy, human connection, resistance to treatment	Not included	Not reported	https://ClinicalTrials.gov/show/NCT02522494
A personalized telehealth intervention for health and weight loss in postpartum women	Weight change	No secondary outcomes	Not reported	https://ClinicalTrials.gov/show/NCT01751230
Evaluation of a carepartner-integrated telehealth rehabilitation program for persons with stroke	Extremity function and use, caregiver conflict, depression	Not included	Not reported	https://ClinicalTrials.gov/show/NCT02703532
Neurocovid rehab and recovery related to COVID-19 diagnosis	Patient health	No secondary outcomes	Not reported	https://ClinicalTrials.gov/show/NCT04638673
Reducing hypoglycemia fear in parents of young children with type 1 diabetes	Hypoglycemia fear	Not included	Not reported	https://ClinicalTrials.gov/show/NCT03879642
Ostomy telehealth for cancer survivors	Patient engagement	Not included	Not reported	https://ClinicalTrials.gov/show/NCT02974634
Telehealth pulmonary rehabilitation for Hispanic and African-American patients admitted with exacerbation of COPD	Composite of COPD hospital readmission/death	Not included	No statistical significant difference was found	https://ClinicalTrials.gov/show/NCT03007485
Vet-harts pilot intervention for veterans with coronary heart disease	QoL, physical limitation, angina stability	No secondary outcomes	Not reported	https://ClinicalTrials.gov/show/NCT01566214
Mobile insulin titration intervention	Optimal insulin dose	Not included	Not reported	https://ClinicalTrials.gov/show/NCT01879579
Evaluation of a prototype diabetes management system applied to insulin initiation and titration	Glycemic control	Time health care providers and subjects spend on managing insulin	No statistical significant difference was found	https://ClinicalTrials.gov/show/NCT01698528
A randomized controlled trial of in-home tele-behavioral health care utilizing behavioral activation for depression	Hopelessness	Psychiatric hospitalizations	No statistical significant difference was found	https://ClinicalTrials.gov/show/NCT01599585
Effects of myofunctional therapy with an application in severe apnea/hypopnea sleep obstructive syndrome (mtassaos)	Number of apneas or hypopneas recorded, blood oxygen level	Not included	Not reported	https://ClinicalTrials.gov/show/NCT04438785
Internet-based motivational interviewing for colonoscopy	Number of participants who completed a screening	Not included	Not reported	https://ClinicalTrials.gov/show/NCT03595904
Optimizing veteran-centered prostate cancer survivorship care	Symptom burden, confidence in self-management	Not included	Not reported	https://ClinicalTrials.gov/show/NCT01900561
Advanced comprehensive diabetes care for veterans with poorly-controlled diabetes	Diabetes control	Not included	Not reported	https://ClinicalTrials.gov/show/NCT01778751
A study of home-delivered neurostimulation for migraine	Migraine days	Not included	Not reported	https://ClinicalTrials.gov/show/NCT03874351
Extension for community healthcare outcomes autism replication evaluation	Autism spectrum disorder screening, conditions correctly treated	Not included	Not reported	https://ClinicalTrials.gov/show/NCT03677089
Cognitive-behavioral conjoint therapy (CBCT) project	Symptom severity, couples satisfaction, psychosocial function, client satisfaction, patient-reported working alliance	Not included	Not reported	https://ClinicalTrials.gov/show/NCT02720016
The effect of the mesentery system on medication adherence	Medication adherence	ED visits, hospitalizations, length of stay	No statistical significant difference was found	https://ClinicalTrials.gov/show/NCT01814696
Improving the frequency and quality of sleep apnea care management	Airway pressure adherence	Not included	Not reported	https://ClinicalTrials.gov/show/NCT01916655
Optimizing dementia care	Caregiver burden, relationship satisfaction, QoL, depression	Use of long-term care facilities	A statistical significant reduction was found in number of hospitalizations and lengths of stay.	https://ClinicalTrials.gov/show/NCT02585232
Mobile technology to engage and link patients and providers in antidepressant treatment	Adherence	Not included	Not reported	https://ClinicalTrials.gov/show/NCT01909973
Behavioral activation therapy for rural veterans with diabetes and depression	Blood glucose levels, depression symptoms	Not included	Not reported	https://ClinicalTrials.gov/show/NCT01572389
Evaluating the CG assist program for caregiving dyads	Activity of daily living	Not included	Not reported	https://ClinicalTrials.gov/show/NCT02021565
Improving sleep in veterans and their CGS	Sleep quality	Not included	Not reported	https://ClinicalTrials.gov/show/NCT02057068
Connect.Parkinson: connecting individuals with Parkinson disease to specialists in their homes	Feasibility of virtual visits, QoL	Minutes spent on the last Parkinson’s disease provider visit	No statistical significant difference was found	https://ClinicalTrials.gov/show/NCT02038959
Telemonitoring after surgery to preserve limb function in optimizing mobility in cancer survivors with skeletal metastases	Inter-rater agreement between video and face-to-face follow up	Not included	Not reported	https://ClinicalTrials.gov/show/NCT02715856
Niclosamide for mild to moderate COVID-19	Respiratory viral clearance	Not included	Not reported	https://ClinicalTrials.gov/show/NCT04399356
Tailored tobacco quitline for rural veterans	Treatment satisfaction	Not included	Not reported	https://ClinicalTrials.gov/show/NCT01592695
Impact of a virtual diabetes self-care and education program on diabetes-related outcomes in Latinos with T2 diabetes	Blood glucose level	No secondary outcomes	Not reported	https://ClinicalTrials.gov/show/NCT02488785
Evaluation of videoconferencing versus telephone genetic counseling	Knowledge retention of genetic counseling information, satisfaction, patient perception, costs	No secondary outcomes	Effect on costs not reported	https://ClinicalTrials.gov/show/NCT02108977
Tailoring treatment targets for early autism intervention in Africa	Joint engagement, child development, socialization, communication	Not included	Not reported	https://clinicaltrials.gov/study/NCT04068688
Telephone based management of hyperlipidemia	Serum level	Not included	Not reported	https://clinicaltrials.gov/study/NCT01212159
Telemedicine control tower for the post-anesthesia care unit	Time to discharge from the post-anesthesia care unit	Not included	No statistical significant difference was found.	https://clinicaltrials.gov/study/NCT04020887
Effectiveness of telephone versus face-to-face CBT in treating people with depression	Number of therapy sessions, number of dropouts, health status, depression	Not included	Telemedicine patients attended statistically significant more sessions	https://clinicaltrials.gov/study/NCT00498706
Veterans telemedicine outreach for PTSD services (VTOPS)	Symptom severity score, health status	Not included	Not reported	https://clinicaltrials.gov/study/NCT00645047
VA cultivating access to resources, education, and skills for dementia caregivers (VA CARES)	Caregiver burden	Not included	Not reported	https://clinicaltrials.gov/study/NCT02106065
Improving obstructive sleep apnea management via wireless telemonitoring	Nightly CPAP adherence	Not included	Not reported	https://clinicaltrials.gov/study/NCT00682838
Telehealth therapy for chronic pain: comparison of in-person versus video-administered act for pain (TTCP)	Pain	Not included	Not reported	https://clinicaltrials.gov/study/NCT01055639
Tobacco cessation for veterans with post traumatic stress disorder (PTSD)	Self-reported quit attempt, progression along the stage of change, prevalence of quitting	Not included	Not reported	https://clinicaltrials.gov/study/NCT00908882
Feasibility of at-home telehealth yoga for treating chronic pain	Satisfaction, adherence, attrition	Not included	Not reported	https://clinicaltrials.gov/study/NCT04074109
Telemedicine strategy with home treatment save resources	Glycemia	Not included	Not reported	https://clinicaltrials.gov/study/NCT02214017
Cardiovascular intervention improvement telemedicine study	Risk of cardiovascular disease	Not included	Not reported	https://clinicaltrials.gov/study/NCT01142908
Practical telemedicine to improve control and engagement for veterans with clinic-refractory diabetes mellitus (PRACTICE-DM)	Hemoglobin A1c	Not included	Not reported	https://clinicaltrials.gov/study/NCT03520413
Telerehabilitation intervention to promote exercise for diabetes	Physical activity	Not included	Not reported	https://clinicaltrials.gov/study/NCT00334113
Early intervention in cystic fibrosis exacerbation (EICE)	Forced expiratory volume	Not included	Not reported	https://clinicaltrials.gov/study/NCT01104402
Prolonged exposure (pe) for post traumatic stress disorder (PTSD): telemedicine versus in person	Treatment completion, PTSD symptoms, depression	Not included	Not reported	https://clinicaltrials.gov/study/NCT01102764
Technology-assisted case management in adults with type 2 diabetes (TACM-DM)	Hemoglobin A1c	Not included	Not reported	https://clinicaltrials.gov/study/NCT01373489
Impact of group motivational interviewing and in-home-messaging-devices for dually diagnosed veterans (GMI-IHMDS)	Alcohol drinking days, alcohol drinks, attendance	Not included	Not reported	https://clinicaltrials.gov/study/NCT00706901
Telehealth-based exercise program to treat fatigue in MS (MS-FIT)	Fatigue	Not included	Not reported	https://clinicaltrials.gov/study/NCT01198977
The PACE/PACENET behavioral health laboratory project (SUSTAINII)	Mental health functioning, caregiver burden	Not included	Not reported	https://clinicaltrials.gov/study/NCT02440594
Carepartner collaborative integrated therapy in sub-acute stroke (CARE-CITE)	Depression, mental health, upper extremity function, stroke impact	Not included	Not reported	https://clinicaltrials.gov/study/NCT04040751
Using telemedicine to improve veteran sleep apnea care	Treatment adherence	Not included	Not reported	https://clinicaltrials.gov/study/NCT01259440
Telephone tinnitus education for patients with traumatic brain injury (TBI)	Changes in tinnitus	Not included	Not reported	https://clinicaltrials.gov/study/NCT01129141
Telemedicine outreach for post traumatic stress in CBOCS (TOP)	PTSD symptom severity	Not included	Not reported	https://clinicaltrials.gov/study/NCT00821678
Relative patient benefits of a hospital-PCMH collaboration within an ACO to improve care transitions	Adverse event	Readmission	No statistical significant difference was found.	https://clinicaltrials.gov/study/NCT02130570
Evaluation of the effectiveness of a telemonitoring program in a cohort of COPD patient with frequent readmissions (TELEPOC)	Hospitalizations	Not included	Telemedicine patients had statistically significant fewer hospitalizations	https://clinicaltrials.gov/study/NCT02528370

### Outcome measures

Of the seventy-eight studies, twenty studies (26 percent) considered clinicians time (nine as primary outcomes, eleven as secondary outcomes, two as both primary and secondary), and four studies measured clinician time directly (one as a primary outcome and three as secondary outcome). The primary outcome measures included the number of admission, readmissions, and length of hospital stay, thus only indirect measures on clinicians’ time. The majority of the studies (66 studies) had clinical measures of morbidity as the primary outcome, and a few were only or in addition to the clinical outcomes focusing on patient satisfaction (8 studies) or adherence (8 studies). Eleven studies included secondary outcome measures related to clinician time, for example, healthcare utilization (2 studies), number of visits to emergency department (1 study), nursing home visits (2 studies), hospitalizations (3 studies), time spent on management of insulin or time spent on provider visits (3 studies).

### Effects on clinicians’ time

Four studies assessed direct measures of clinicians’ time (Table 2), but no statistically significant difference between telemedicine versus usual care groups was found. Of the sixteen studies assessing the indirect effects of telemedicine on clinicians’ time, thirteen found no significant effects. Two studies (https://ClinicalTrials.gov/show/NCT02585232 and https://clinicaltrials.gov/study/NCT02528370) found statistically significant fewer hospitalizations favoring the telemedicine arm, and one trial found that patients in the telemedicine arm attended statistically significant more therapy sessions (https://clinicaltrials.gov/study/NCT00498706).

**Table 2.** Records assessing direct measures of time needed to treat

Title	Outcome measure	Result		URL
		Central tendency	Variability	
Evaluation of a prototype diabetes management system applied to insulin initiation and titration	Number of minutes that clinicians and patients spend on managing insulin titration through appointments, phone calls, emails, or faxes	Mean number of minutes per patient: intervention group (108); control group (80)	Stand deviation: intervention group (41); control group (51)	https://clinicaltrials.gov/ct2/show/NCT01698528
Connect.Parkinson: connecting individuals with Parkinson disease to specialists in their homes	Duration of time spent with the physician during in-person or telemedicine care	Median number of minutes per patient: intervention group (30); control group (30)	Inter quartile range: intervention group (20–40); control group (20–45)	https://clinicaltrials.gov/ct2/show/results/NCT02038959
Utilizing telemedicine for delivery of postoperative care (telemedicine)	Number of minutes for the postoperative encounter by telemedicine or a traditional visit	Mean number of minutes per patient: intervention group (9.1); control group (9.2)	Standard deviation: intervention group (4.0); control group (2.6)	https://clinicaltrials.gov/ct2/show/NCT04348357
Telemedicine control tower for the post-anesthesia care unit	Number of minutes for the patient to be ready for discharge from the post-anesthesia care unit	Median number of minutes per patient: intervention group (91.5); control group (84.5)	Inter quartile range: intervention group (62–145); control group (54–116.5)	https://clinicaltrials.gov/study/NCT0402088

## Discussion

Health systems globally struggle with a shortage of clinician time, caused by a lack of healthcare workers and an increased demand for healthcare in the population. Telemedicine (and other digital solutions) may contribute to solving this problem by making care more time-efficient. However, we found that randomized controlled trials and observational studies of telemedicine from the last 10 years generally did not assess whether telemedicine saved clinicians’ time compared to usual care – or not. When assessed, clinician time was usually only considered indirectly. The few studies that measured clinician time directly showed no statistically significant difference, while only three out of sixteen studies measuring indirect assessments showed that telemedicine resulted in either a statistically significant reduction in the use of clinician time (two trials) or a statistically significant increased number of therapy sessions per patient (one trial).

It should be noted that not all telemedicine services are expected to have an impact on clinicians’ use of time. Therefore, not all randomized controlled trials or observational studies should include this as a primary or secondary outcome. Some digital interventions may simply replace a face-to-face meeting with a digital one without affecting the duration of the contact. Hypothetically, it may also be the case that telemedicine increases clinician time needed if loads of data need to be interpreted for healthy patients. As an example, a recent study of the Apple Watch has described the potential problem of false positive screening results leading to overutilization of healthcare resources (19).

In addition, telemedicine may also lead to increased access to care i.e., a larger proportion of the population eligible for the intervention or a lower no-show rate (20). Thereby telemedicine may require more clinician time than usual care. It is however important to keep in mind that telemedicine can have several objectives: to improve quality of care, to increase access to care, to save time for patients, and/or to save time for clinicians. If telemedicine is found to improve the quality of care, an increased demand for clinician time compared to usual care may very well be warranted.

### Strengths and weaknesses

Our review is strengthened by the systematic search for randomized trials or observational studies with a comparison group registered in the last decade, a choice made to potentially include the most reliable and pertinent evaluations. We may have missed registrations as we only searched using “telemedicine” as the search term. Combinations with other search terms such as “telehealth,” “digital health,” “home monitoring,” and so forth would have resulted in more studies and a more comprehensive review.

The results found in this review need to be validated by more traditional systematic reviews of published studies of the effect of telemedicine on clinicians’ time, for example, by reviewing studies in the MEDLINE, EMBASE, Cochrane database of systematic reviews and so forth Such a review could also find information of changes in the primary and secondary outcomes that is not included in the protocol in clinicaltrials.gov.

### Implications

Current guidance for HTA and health economic evaluation of telemedicine recommends assessing the impact of telemedicine interventions on resources and workflow (13;17). Assessing “clinician time” in studies and HTA analyses of telemedicine has several advantages. It could help avoid implementation of telemedicine interventions that would increase the demand on clinician time in an unreasonable way, and it could increase the implementation of effective interventions by enhancing acceptability among stressed clinicians.

Assessment of clinicians’ time could be done by direct or indirect measures of time. Examples of direct measures could be number of minutes spent by clinicians on implementation of the telemedicine service and number of minutes per patient spent by clinicians when providing the telemedicine service. Importantly, such measures also have to take into account if the telemedicine intervention expands the population eligible for the intervention compared to usual care. Examples of indirect measures could be number of admissions per patient, length of stay per admission, number of hospital outpatient visits per patient, emergency department visits, phone calls, video consultations per patient, number of visits to general practice per patient, and number of home healthcare visits per patient.

Both randomized controlled trials (RCTs) and observational studies will be valuable in future investigations into the impact of telemedicine on clinicians’ time utilization. While the former design offers the advantage of high internal validity, the latter may better capture effects on clinician time in real-world settings, enhancing external validity. However, observational before-after studies may be susceptible to confounding bias, such as that caused by other changes in staff or healthcare organization over time, and measurements may be less standardized compared to a randomized controlled trial. A potential solution to address these challenges is to design future studies on the effects of clinician time as cluster randomized trials. In this approach, for example, entire clinical departments and their staff are randomized to either telemedicine or usual care (21). This design aims to mitigate confounding factors and enhance the robustness of the findings.

Information about these direct and indirect measures could be collected as patient-level data in clinical studies and HTA by observation of healthcare professionals providing the telemedicine service, by means of digital registration systems (22), by interviews or surveys with healthcare personnel (23), or by use of data from electronic health records (EHR) (24).

If the recommendation by Johansson et al. (12) is followed and future studies of telemedicine increase the inclusion of effects on clinician time as primary or secondary outcomes, this may also have implications for the design and content of the telemedicine interventions. Even though it is usual practice to let the primary outcome be defined by the objective and design of the study (25), it is possible that the relation could also be the other way around – that defining clinicians’ time as a primary or secondary outcome may have an impact on the expectations of the effects of the intervention and thereby on the design and the content of the intervention itself. In the process of developing a telemedicine intervention you need to make many choices, because telemedicine often consists of several components. You need to find the right digital communication technology, the right patient group, the right clinicians to use the technology, the right level of integration with other IT systems and so forth. Telemedicine interventions have therefore been described as complex interventions (26). Thus, a telemedicine intervention can be developed and designed in many ways depending on the objective. Placing priority on measuring clinicians’ time as an outcome of interest should affect how investigators design and conduct effective studies. Furthermore, consensus about the need to assess the effect of interventions on TNT could contribute to reduce the demands on clinician time encoded in the design of the interventions themselves. For instance, such a consensus could drive the development of digital health solutions that rely on automatically measured or patient-reported measures and on algorithms to determine which patients need clinician time.

## Conclusion

In conclusion, the review of seventy-eight protocols from studies of telemedicine found that four studies included direct measures of clinician time, and sixteen studies included indirect measures, for example, readmissions and length of hospital stay. Assessment of effects on clinician time in clinical studies and in HTA of telemedicine may increase the potential of telemedicine to help solve one of the major challenges for health systems today – a shortage of clinician time caused by a lack of healthcare workers occurring in tandem with an increased demand of healthcare in the population.

## Key points

- This scoping review found that clinician time is not commonly measured in randomized trials or observational studies of telemedicine interventions.
- Attention to clinician time in evaluations of telemedicine may bring attention to the organization of workflows and increase the value of telemedicine.
